# Feasibility of Telephone Follow-Up after Critical Care Discharge

**DOI:** 10.3390/medsci8010016

**Published:** 2020-03-14

**Authors:** Sofia Hodalova, Sarah Moore, Joanne Dowds, Niamh Murphy, Ignacio Martin-Loeches, Julie Broderick

**Affiliations:** 1 Discipline of Physiotherapy, School of Medicine, Trinity College Dublin, the University of Dublin, Dublin D08 W9RT, Ireland; hodalovs@tcd.ie (S.H.); julie.broderick@tcd.ie (J.B.); 2 Department of Physiotherapy, St James’s Hospital, Dublin D08 X4RX, Ireland; samoore@stjames.ie (S.M.); jdowds@stjames.ie (J.D.); nimurphy@stjames.ie (N.M.); 3 Department of Anaesthesia and Critical Care Medicine, St James’s Hospital, Dublin D08 X4RX, Ireland; 4 Multidisciplinary Intensive Care Research Organization (MICRO), St James’s Hospital, Dublin D08 X4RX, Ireland; 5 Pulmonary Intensive Care Unit, Respiratory Institute, Hospital Clinic of Barcelona, IDIBAPS, University of Barcelona, CIBERes, 08036 Barcelona, Spain

**Keywords:** frailty, quality of life, recovery, intensive care, critical care, telephone

## Abstract

**Background:** Critical care has evolved from a primary focus on short-term survival, with greater attention being placed on longer-term health care outcomes. It is not known how best to implement follow-up after critical care discharge. Study aims were to (1) assess the uptake and feasibility of telephone follow-up after a critical care stay and (2) profile overall physical status and recovery during the sub-acute recovery period using a telephone follow-up assessment. **Methods:** Adults who had been admitted to critical care units of St. James’s Hospital, Dublin, for >72 h were followed up by telephone 3–9 months post discharge from critical care. The telephone assessment consisted of a battery of questionnaires (including the SF-36 questionnaire and the Clinical Frailty Scale) and examined quality of life, frailty, employment status, and feasibility of telephone follow-up. **Results:** Sixty five percent (*n* = 91) of eligible participants were reachable by telephone. Of these, 80% (*n* = 73) participated in data collection. Only 7% (*n* = 5) expressed a preference for face-to-face hospital-based follow-up as opposed to telephone follow-up. For the SF-36, scores were lower in a number of physical health domains as compared to population norms. Frailty increased in 43.2% (*n* = 32) of participants compared to pre-admission status. Two-thirds (*n* = 48) reported being >70% physically recovered. **Conclusion:** Results showed that telephone follow-up is a useful contact method for a typically hard-to-reach population. Deficits in physical health and frailty were noted in the sub-acute period after discharge from critical care.

## 1. Introduction

Advances in critical care treatment have improved mortality rates, and thus the focus has moved from short-term survival towards the “new frontier” [1] of addressing longer-term health consequences [2] in critical care survivors. Although survival prospects have improved, post-intensive care syndrome (PICS), which is characterised by persistent cognitive, physical, and functional impairments, is commonly experienced after surviving the acute phase of critical illness [3,4,5].

Clearly there is a need for improved discharge planning and follow-up care [6,7]. The subacute recovery phase may be an opportune time to follow up patients, as during the first six months after an intensive care (ICU) stay, healthcare utilization is increased, with almost half (46%) of all individuals admitted to an emergency department and nearly one-third (29%) re-hospitalized [8].

In 2017, the United Kingdom’s National Institute for Health and Care Excellence (NICE) recommended funding assessment and provision of a rehabilitation plan for survivors of critical illness [9]. Even though ICU follow-up clinics generally do not offer demonstrable benefits in terms of health-related or economic outcomes [10], less tangible patient-related benefits clearly pertain to critical care follow-up [11,12].

The challenge remains to elucidate the optimum method of follow-up from critical care. The aim of the present study was twofold: (1) to determine the feasibility of telephone follow-up after a critical care stay; and (2) to investigate self-reported physical performance variables, quality of life, frailty, return to work, and physical recovery 3–9 months after discharge from critical care using a telephone follow-up assessment.

## 2. Methods

### 2.1. Study Design

This cross-sectional study evaluated patients discharged from critical care in St. James’ Hospital Dublin at a single time point between 3 and 9 months post discharge from hospital. Critical care consisted of the ICU and high-dependency unit (HDU) in this setting. Ethical consent was approved for this study by the Tallaght University Hospital/St James’s Hospital Joint Research Ethics Committee (REC).

### 2.2. Study Procedure

The study took place in a large acute hospital and tertiary referral centre. Using the computerised hospital data management system, all those admitted to critical care beds from November 2017 to April 2018 were screened for suitability. Consideration for study enrolment was based on the following criteria: age > 18 years, stay in critical care longer than 72 h, documented communication or cognitive difficulties which would preclude ability to participate in telephone interview, death post discharge from hospital, lack of a direct contact number, current inpatient status, or any documented reason which could suggest an inability to participate in a telephone interview, such as dementia.

### 2.3. Data Collection

Following the screening process, each identified potential participant was contacted via a telephone call for a maximum of three call attempts. At least one telephone attempt took place in the morning and afternoon. If the potential participant was not reached in a third telephone call attempt they were subsequently excluded from the study.

During the contact telephone call, information was provided about the study with the opportunity for questions or concerns to be addressed. If the potential participant expressed no interest in study involvement, no further contact was made. If the potential participant displayed interest, an information leaflet and consent form were posted to their home address with the understanding that a second telephone call would be undertaken for verbal consent and the telephone assessment. The second telephone call was made at least one week after the initial call to confirm agreement to participate and to undertake the telephone follow-up assessment.

The verbal consent and telephone-based assessment took approximately 20 min. The telephone assessment consisted of a battery of questionnaires examining quality of life, frailty, employment status, and feasibility of telephone follow-up.

### 2.4. Study Measures

#### 2.4.1. Health-Related Quality of Life (HRQoL)

HRQoL was measured using the SF-36. The SF-36 is a 36-item questionnaire that measures eight domains of health: physical functioning, physical role, disability due to physical health, pain, general health perceptions, vitality and social functioning, emotional problems, and mental health. Scoring is from 0 to 100, with 0 indicating the worst health state and 100 the best [13].

#### 2.4.2. Frailty

The Clinical Frailty Score (CFS) was used to assess frailty. The CFS was adapted from the clinical frailty scale [14] and contains nine categories from very fit to terminally ill. The categories correspond to the following: 1 (very fit), 2 (well), 3 (managing well), 4 (vulnerable), 5 (mildly frail), 6 (moderately frail), 7 (severely fail), 8 (very severely frail), and 9 (terminally ill) [15]. Participants were considered to be frail when the CFS was ≥ 5 [16]. Participants were asked to rate the CFS at the current time and pre-admission.

#### 2.4.3. Other Outcomes

Participants were asked to self-rate physical recovery post discharge between 0 and 100%, with 0% corresponding to no perceived recovery and 100% considered a return to previous levels of physical fitness, in response to the question “to what extent do you feel physically recovered” based on the work of Onerup et al. (2015) [17]. Employment status in terms of working or being retired or unemployed before and after hospitalisation was also recorded. Their opinion on the feasibility of a telephone call was also obtained, with a preference (if any) for either a telephone call or hospital appointment as a means for a single assessment method. Participants were advised to access their General/Medical Practitioner for follow-up care if necessary.

### 2.5. Statistical Analysis

Data were collected in a database and analysis was performed using IBM SPSS Statistics 24. Patient characteristics were summarised descriptively. Polar plots were generated to demonstrate comparison of SF-36 results to normal normative datasets from the United States [18], the United Kingdom [19], France [20], and Norway [21]. To assess frailty before hospital admission and at the time of telephone follow-up, data were first assessed for normality. As data were normally distributed, a paired *t*-test was used to compare frailty between the two time points, with *p* < 0.05 considered statistically significant.

## 3. Results

**Figure 1:**
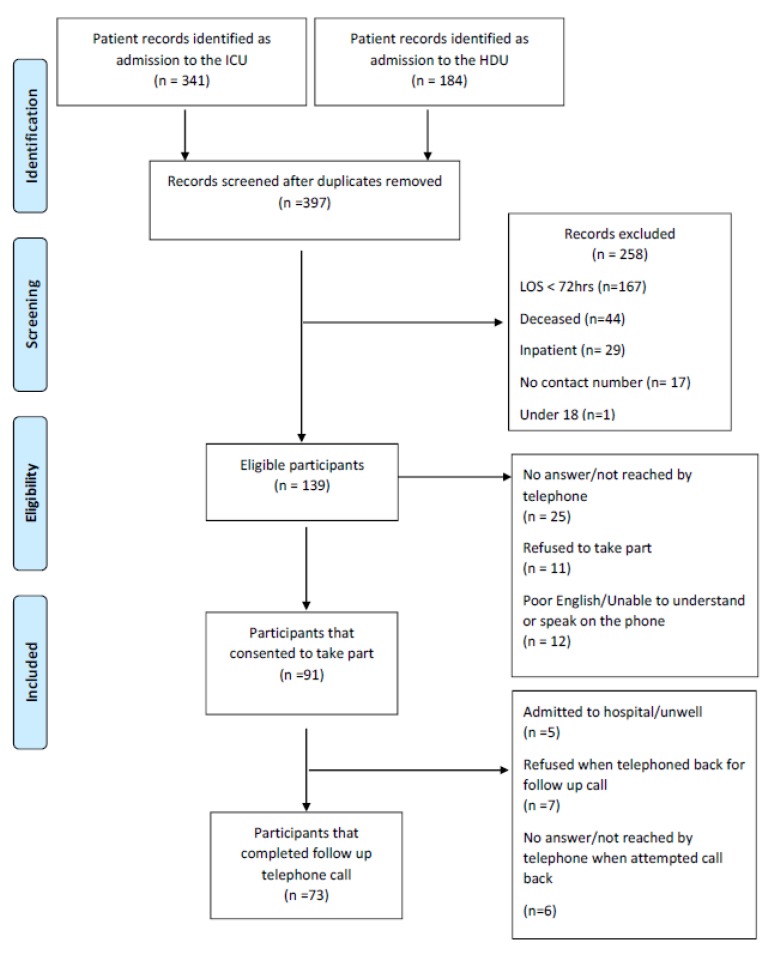
Flow of participants through the study. ICU: Intensive care unit, HDU: High-dependency unit.

Data collection for this study took place from 25 June to 17 August 2018. In total, 525 patient records of admission to the ICU/HDU were recorded on ICU and HDU databases. After removal of duplicates, 397 patient records were identified. Inclusion criteria were met by 139 of these patients who were each contacted. Initially, 91 patients were interested in receiving further information about this study and information leaflets were sent to their preferred address via the postal system. Of these, 73 agreed to study participation and completed the final follow-up call. The flow of participants through the study is shown in Figure 1.

Participant characteristics are shown in Table 1. The mean (SD) age of all patients was 61.7 (15.7) years, with a range of 26 to 90 years. Gender was fairly evenly distributed, with 55% males (*n* = 40) and 45% females (*n* = 33). The average length of stay in critical care (ICU and HDU combined) was 8 days. The mean (SD) total length of stay from admission to discharge from hospital was 34 (24.4) days. Reason for admission to critical care are also noted in Table 1. Eleven percent (*n* = 44) died since critical care discharge.

**Figure 2:**
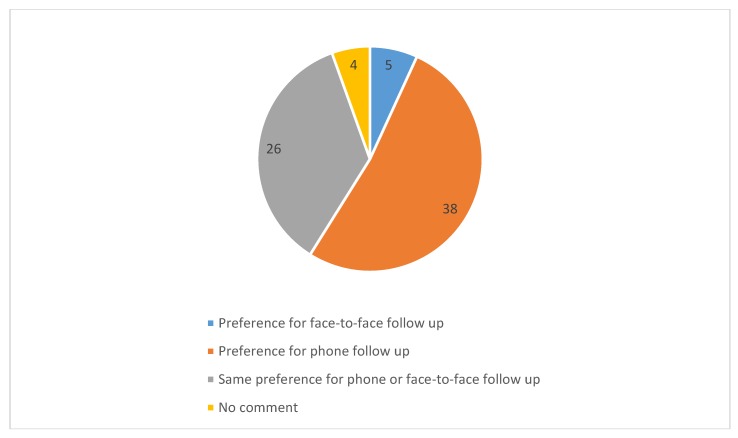
Pie chart of phone versus face-to-face person follow-up preference, participant numbers included.

Of the eligible participants, 22% (*n* = 31) did not answer or were not reachable by telephone. Fewer than 10% (8.6%, *n* = 12) had a language or cognitive barrier which precluded participation in the telephone assessments. There was a low refusal rate of 13% (*n* = 18). Participant perspective on the feasibility of a telephone call as means of a follow-up tool was also recorded, as shown in Figure 2. Over half (52%, *n* = 38) expressed a preference for telephone follow-up, 7% (*n* = 5) expressed a preference for in person hospital based follow-up, and 35.6% (*n* = 26) did not express any preference.

**Figure 3:**
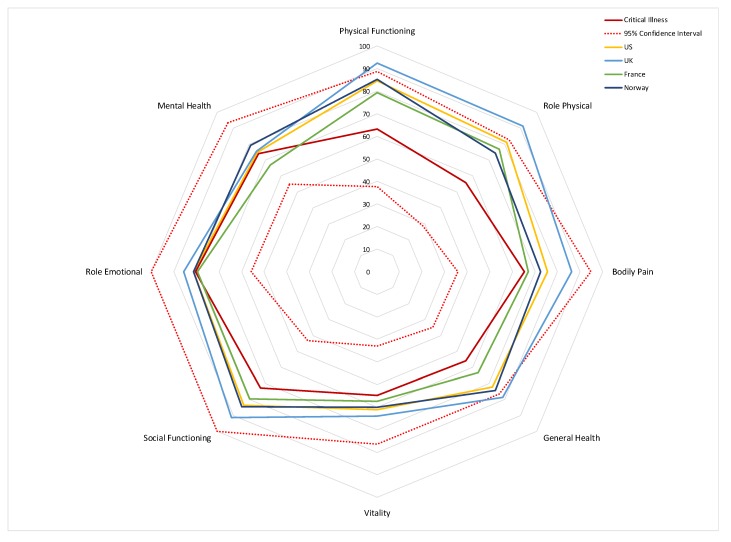
Polar plot HRQoL (SF-36) values for participants (solid red line) compared to population norms from the United States, the United Kingdom, France, and Norway. The confidence interval is indicted by the broken red line (confidence interval of study participants is the inner red broken line, confidence interval of populations normative values is the outer broken red line).

Mean (SD) domain scores for the HRQoL instrument (SF-36) are shown in Table 2. Lowest scores for HRQoL were noted for domains of physical functioning, role physical, general health, and vitality. Mean SF-36 scores in this study sample are shown compared to normative scores of healthy individuals in Figure 3. The figure shows that this cohort after critical care appears to have lower HRQoL than the normal population, particularly in relation to physical domains (physical functioning and role functioning) and general health. Data for 2 participants were missing for the SF-36 due to difficulty in understanding the questionnaire.

**Figure 4:**
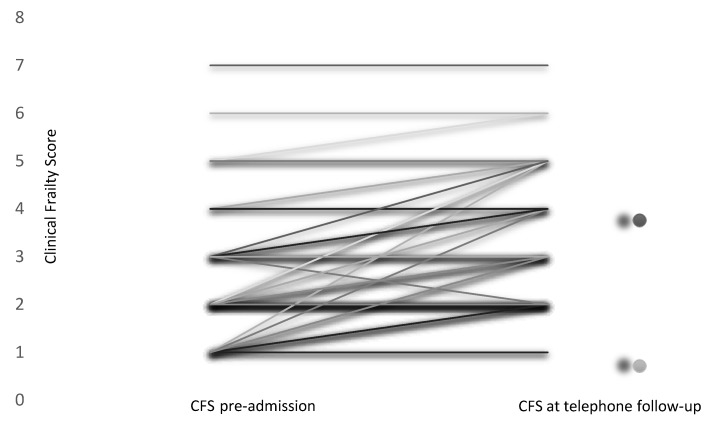
CFS estimated pre-admission to critical care and current status (3–9 months after hospital discharge). CFS: Clinical Frailty Score.

CFS scores ranged from 1 (classified as very fit for their age) to 7 (severely frail). Estimated frailty pre-admission and at the time of the telephone interview is shown in Figure 4. Eight percent (*n* = 6) were considered frail pre-admission, which increased to just under 20% (*n* = 14) at the time of telephone follow-up—a significant increase in frailty (*p* < 0.001). Estimated mean (SD) pre-admission frailty was 2.44 (1.3) and this value was 3.1 (1.3) at telephone follow-up 3–9 months after discharge from critical care. Just over half of participants (54.8%, *n* = 40), reported the same level of frailty at the time of the telephone interview compared to their pre-admission status. Frailty increased in 43.2% (*n* = 32) of participants and decreased in only one participant from pre-admission to the time of telephone follow-up.

Percentage of physical recovery was noted as the patient’s interpretation of their physical health at the time of telephone assessment. This percentage ranged from 20% to 100%. Two-thirds (66%, *n* = 48) reported they were >70% physically recovered. Data from this question were missing for eight participants (*n* = 11%) due to difficulty rating their own recovery or misunderstanding the question. Fifteen percent (*n* = 11) were 50%–70% recovered, and 8.2% (*n* = 6) were <50% recovered. Anecdotally, the most commonly mentioned factor impeding physical recovery was low energy due to often feeling tired/fatigued.

As previously stated, 53% (38) of participants were under the retirement age of 66. It was noted that half of this population (*n* = 24), reported working before their admission. Out of these, 13 (54%) had returned to work since discharge. Only 4 (30%) reported returning to the same level of work as pre-admission. The rest of the participants (*n* = 47), reported being either retired or not working before their admission to critical care.

## 4. Discussion

Overall this study demonstrated the feasibility of telephone follow-up in critical care survivors. High satisfaction levels were recorded for telephone follow-up. The telephone interview indicated that the majority of participants had not returned to baseline levels of physical health and over 40% reported increased frailty compared to their pre-admission status.

Telephone follow-up may potentially be useful to screen for “high risk” patients who require follow-up and provide post-discharge care, although this method of contact did not imply access to all participants. From 139 participants that were eligible to participate, full data were obtained for just over half of participants. Telephone follow-up was achieved in 51% of individuals in one population after mild traumatic brain injury [22] and 36% in another population after an emergency department visit [23]. The reason for the lack of contact in 18% (*n* = 25) of eligible participants is unknown. Potentially they were too unwell to take a telephone call or perhaps at the other end of the spectrum were back at work and missed the call. There was a low refusal rate of 8% (*n* = 11) and 9% (*n* = 12) had poor English or were unable to speak on the telephone.

We were unable to collect data from 20% (*n* = 18) of potential participants who had initially consented to study participation, due to refusal/change of mind when contact was made again (*n* = 7), being unable to make phone contact again despite repeat attempts (*n* = 5), or because of readmission to hospital/unwellness (*n* = 5).

Slightly more males (55%) than females (45%) participated in this study. There was a diversity in ages, with just over half (53%) of participants under the age of 66 years. The average length of stay in critical care was 8 days, while the total hospital stay was 34 days. As the study site is a national referral centre for many specialised cancer-related surgeries such as oesophagectomies and head and neck surgeries, transfer from critical care to tertiary hospitals for recovery may have artificially shortened the average admission time in critical care. The specialised nature of surgeries conducted in this site is reflected in that almost half of participants (47%) were admitted for post-operative management and monitoring and only a small proportion (12%) were admitted for reasons such as trauma.

Theoretically, the recovery trajectory of people after surgery would be expected to be faster compared to for instance multiple trauma due to accidents, which accounted for a low proportion of patients in critical care. Despite this, physical health domains of the quality of life measure were lower than normative values, which mirrors one-year follow-up data in critical care survivors [24,25]. This is backed up by clinical frailty data which showed that almost 40% of participants displayed higher levels of frailty than pre-admission. This is a potentially worrying physical health trajectory, suggesting that in the sub-acute period of discharge from critical care, physical rehabilitation needs persist. Longitudinal studies should explore if and when physical status returns to baseline, and whether implementation of an appropriate physical intervention or rehabilitation strategies may hasten the return to full physical health.

Perhaps at odds with reported physical health and frailty, two-thirds of participants optimistically reported themselves to be >70% physically recovered. This discordance between a somewhat arbitrary composite measure of how physically recovered participants “feel” and actually reported frailty and physical health may not be surprising. It is perhaps reflective that even with low levels of physical activity and physical performance activities of daily living can be conducted with relative ease. This may also be related to patient expectations and is perhaps how physical recovery is self-assessed and likely cannot be considered a true comparison to baseline levels of activity and performance.

Of those who worked pre-admission, over half (54%) were reported to be back at work, similar to values of 50% in another critical care population [24,25]. This is perhaps indicative of fatigue and low energy levels which were commonly cited reasons for not achieving full physical recovery.

It appears most intensive rehabilitation programmes post critical care [26,27,28,29,30,31] fail to show tangible benefits in outcomes such as quality of life, functional outcomes or recovery. This may be partly due to the inability to identify and target high-risk groups [1] so the feasibility of a telephone follow-up for critical care survivors demonstrated in this study deserves consideration in this typically hard to reach population.

Nonetheless, as PICS is a multi-dimensional construct with no gold standard method of measurement, it is difficult to assess how successfully this method of follow-up assessed its presence. Advantages of telephone follow-up are that it is simple to implement, low-cost, and can increase patient satisfaction [32,33,34]. The telephone battery employed in this study was feasible and for the most part easily conducted over the telephone. Despite potential issues with self-report bias, telephone follow-up could be considered a useful screening or triage tool and at risk individuals for PICS could then be signposted to more intensive services.

The self-report nature of all measures (frailty, health-related quality of life, and physical recovery) employed in this study is an inherent limitation of telephone follow-up. A further limitation was the single-centre design. It is not known how applicable this critical care population is to other settings. There is a “culture” of early mobilisation within critical care in this setting, so typically where possible all participants would have been mobilised as early as safely possible. An Enhanced Recovery After Surgery (ERAS) protocol including early mobilisation was in place, relevant to a number of surgical patient groups including those with oesophagectomies. Clinical status at hospital discharge was not recorded in a consistent manner in this setting or collected specifically for the purpose of this study. Based on the CFS scores reported in this study it is likely that participants were discharged with a diverse range of physical abilities. Pre-existing frailty or other co-morbid conditions were not considered for this study and may have influenced physical status independent of their critical care stay.

While telephone follow-up may be an underutilized tool to access a significant proportion after a critical care stay, the cohort we were unable to reach deserves careful consideration. It would be useful to assess if a combination of in-person contact before discharge from hospital as well as telephone follow-up would capture a larger proportion of critical care survivors to minimize non-response bias. Future studies should also elucidate cut-off points for follow-up and the most useful outcomes to incorporate, as well as content and timing of telephone calls. Incorporating the triage system devised by Vijayaraghavan [1] may be useful so people with lower acuity illness and hospital exposure may not need to be contacted, but this requires further evaluation. Telephone assessment should also be verified by face-to-face objective assessment for accuracy. Virtual clinics represent an evolving field, using existing communication technology to improve access and acceptability for healthcare assessments. A recent study showed that in a population with advanced kidney disease, a virtual programme of multi-disciplinary care was feasible and generated a high satisfaction rate from participants [35]. While this patient group in this research study would not be considered digital natives it is unfair to assume they are not technologically proficient. With appropriate access to technology and equipment this could also be a way of monitoring recovery in patients post critical illness, which may overcome previously reported concerns for patients regarding distance and returning to a healthcare institution associated with negative life events.

Results of this study raise the questions of whether physical and mental recovery take longer than the 3–9 month time-line after critical care discharge employed in this study, whether this process happens naturally, and whether rehabilitation strategies are needed to optimise health so baseline levels of functioning are reached. Future research should also explore the conundrum between self-perceived “physical recovery” and reported physical health and frailty to ensure optimal outcomes and rehabilitation strategies are implemented for this growing population of critical care survivors.

## 5. Conclusions

This study indicated the feasibility of telephone follow-up after critical care which could be used to screen for “at risk” patients who require signposting to follow-up services after a critical care stay. The telephone interview highlighted physical health deficits and frailty at this time-point post critical care discharge. Telephone follow-up may be an underused tool to screen patients after critical care, and should be evaluated in further studies.

## Figures and Tables

**Table 1:** Participant characteristics.

Variable	*n* = 73
Female, *n* (%)	33 (45)
Age, mean years (SD)	61.7 (15.7)
LOS critical care, mean days (SD)	8 (5.5)
LOS hospital, mean days (SD)	34 (24.4)
**Reason for admission to critical care, *n* (%)**	
Post-operative follow-up	34 (47)
Respiratory-related complications sepsis, MOF	24 (33)
Kidney-related complications	6 (8)
Trauma, epilepsy, other	9 (12)

MOF: multiple organ failure, LOS: length of stay.

**Table 2:** Mean (SD) HRQoL(SF-36) domain scores.

Domain	*n* = 71
Physical functioning	63.3 (25.5)
Role physical	55.7 (27.0)
General health	55.8 (20.9)
Vitality	54.7 (21.8)
Social functioning	72.9 (30.0)
Role emotional	80.4 (24.6)
Mental health	74.1 (19.3)
